# Identifying genetically redundant accessions in the world’s largest cassava collection

**DOI:** 10.3389/fpls.2023.1338377

**Published:** 2024-01-18

**Authors:** Monica Carvajal-Yepes, Jessica A. Ospina, Ericson Aranzales, Monica Velez-Tobon, Miguel Correa Abondano, Norma Constanza Manrique-Carpintero, Peter Wenzl

**Affiliations:** Genetic Resources Program, Alliance Bioversity International and International Center for Tropical Agriculture (CIAT), Cali, Colombia

**Keywords:** cassava, genebank, genetic redundancy, curators, diversity

## Abstract

Crop diversity conserved in genebanks facilitates the development of superior varieties, improving yields, nutrition, adaptation to climate change and resilience against pests and diseases. Cassava (*Manihot esculenta*) plays a vital role in providing carbohydrates to approximately 500 million people in Africa and other continents. The International Center for Tropical Agriculture (CIAT) conserves the largest global cassava collection, housing 5,963 accessions of cultivated cassava and wild relatives within its genebank. Efficient genebank management requires identifying and eliminating genetic redundancy within collections. In this study, we optimized the identification of genetic redundancy in CIAT’s cassava genebank, applying empirical distance thresholds, and using two types of molecular markers (single-nucleotide polymorphism (SNP) and SilicoDArT) on 5,302 *Manihot esculenta* accessions. A series of quality filters were applied to select the most informative and high-quality markers and to exclude low-quality DNA samples. The analysis identified a total of 2,518 and 2,526 (47 percent) distinct genotypes represented by 1 to 87 accessions each, using SNP or SilicoDArT markers, respectively. A total of 2,776 (SNP) and 2,785 (SilicoDArT) accessions were part of accession clusters with up to 87 accessions. Comparing passport and historical characterization data, such as pulp color and leaf characteristic, we reviewed clusters of genetically redundant accessions. This study provides valuable guidance to genebank curators in defining minimum genetic-distance thresholds to assess redundancy within collections. It aids in identifying a subset of genetically distinct accessions, prioritizing collection management activities such as cryopreservation and provides insights for follow-up studies in the field, potentially leading to removal of duplicate accessions.

## 1 Introduction

In the 1960s, plant scientists began coordinated collection and conservation efforts to reverse the decline of traditional landraces of essential food crops associated with the Green Revolution. After more than six decades, they have preserved over seven million germplasm accessions from more than 16,500 plant species (FAO, 2010). These invaluable genetic resources are now safeguarded in 1,750 genebanks worldwide (Engels, 2003; FAO, 2010). Most of these plant samples are conserved away from their natural habitats, forming ex-situ collections (Cohen et al., 1991). These collections play a crucial role in developing superior crop varieties, enhancing yields, improving nutrition, adapting to climate change, and bolstering resilience against pests and diseases (Westengen et al., 2018; Sheat et al., 2019; Baptista et al., 2022; Mba and Ogbonnaya, 2022). They therefore contribute to enhancing agriculture, bolstering food security, and sustaining livelihoods.

Cassava (*Manihot esculenta* Crantz) is believed to have been domesticated in the Amazon Basin over 6,000 years ago (Olsen and Schaal, 1999; Olsen, 2004; Carvalho et al., 2017). Cassava provides nutrition to more than 500 million people in Africa and other continents around the world (Howeler et al., 2013). Presently, there are more than 13,832 cassava accessions conserved ex situ in genebanks across at least nine countries globally. Colombia, Brazil, and Nigeria are the countries conserving the largest collections with 5,963, 3,620, and 3,234 accessions, respectively (genesys-pgr.org/,Genesys, 2023). Colombia hosts an international genebank at the International Center for Tropical Agriculture (CIAT) that conserves the world’s largest *in-vitro* collection of cassava and its wild relatives. The collection consists of 5,577 accessions of the cultivated species and 386 wild relatives belonging to 23 *Manihot* species from south-western North America to northern Argentina (Allem, 1994; Duputié et al., 2011). The cultivated species can be propagated either by seed or vegetatively from stems, the latter being the most common practice for commercial production (Howeler et al., 2013). Vegetative propagation, ensures that the new plants are genetically identical to the parent plant, preserving desired traits such as disease resistance, high yield, and nutritional quality in the next generation (ibid).

In most crop species, true seeds serve as the primary method for germplasm conservation (Engels, 2003). However, cassava is a highly heterozygous and clonally propagated crop and cannot rely on true seeds for conservation efforts (Mafla et al., 1993; Qi et al., 2022). Until the 1990s, CIAT’s cassava germplasm collection was maintained both in the field and *in-vitro* (Mafla et al., 1993). However, due to increasing difficulties in managing the field collection, only the *in-vitro* collection was retained under slow-growth conditions (Hershey, 2008). *In-vitro* cultures serve as sources of disease-free materials for distribution, multiplication, and as explants for cryo-preservation (FAO, 2014).

Conserving the cassava collection *in-vitro* is costly. A 2011 study estimated a conservation cost of US$71 per accession, which in 2023 would be equivalent to US$97 (CGIAR Genebanks Consortium 2011). Considering that plant genetic resources for food and agriculture are conserved in perpetuity, identifying genetically redundant accessions or potential duplicates within the collection is crucial for optimizing physical storage, reducing maintenance costs, enhancing characterization, and ensuring collections’ accessibility and usability.

Over the past decades, various methods have been employed to differentiate between genotypes in genebank collections. Initially, biochemical markers were employed (Lefèvre and Charrier, 1992), followed by the use of molecular markers such as random amplified polymorphic DNA, amplified fragment length polymorphism, and microsatellites (Virk et al., 1995; Dean et al., 1999; Kisha and Cramer, 2011; Motilal et al., 2013). In recent years, the advent of high-throughput sequencing technologies has revolutionized the characterization of genebank crop collections, such as wheat, barley, bean, cassava, and rice (Nadeem et al., 2018; Ferguson et al., 2019; Milner et al., 2019; Sansaloni et al., 2020; Tanaka et al., 2021). These techniques examine genome-wide natural variation patterns (Orek et al., 2023) and enable the comprehensive assessment of genetic distinctness and redundancy across entire genomes (Fu, 2023; Orek et al., 2023). The genetic distinctness and redundancy of cassava clones grown by farmers or conserved in genebanks has been evaluated using a variety of methods, revealing genetic redundancy levels of 20−50 percent within and across collections (Chavarriaga-Aguirre et al., 1999; Albuquerque et al., 2019; Orek et al., 2023; Soro et al., 2023).

We hypothesize that establishing empirically defined genetic-distance thresholds will enable the effective identification of genetically redundant accessions within the vast cassava collection at CIAT. The aim of this study was thus to (a) empirically define genetic-distance thresholds to identify genetically redundant accessions and (b) identify genetically redundant accessions within the largest global cassava collection conserved at CIAT. The results of this study provide valuable insights for efficient collection management, while generating genetic characterization data that will enable a more targeted use of accessions supporting cassava crop improvement for the current and future crop challenges.

## 2 Methods

### 2.1 Plant materials and genebank accessions

A subset of 21 accessions were randomly selected from the CIAT cassava core collection (core collection defined by Hershey et al., 1994) conserved in Palmira, Colombia, to establish the thresholds for identifying genetically redundant accessions. Leaf tissue was collected from plantlets conserved *in vitro* under slow-growing conditions to extract DNA, generating various biological and technical replicates. These replicates included: (i) different individuals from the same accession obtained from different conservation units, referred to here as “Ind-Reps”; (ii) distinct DNA samples extracted from the same individual, referred to here as “Extract-Reps”, and (iii) the same DNA sample analyzed twice, labeled as “DNA-Reps” (**Supplementary Figure 1**). Among the 21 accessions, a total of 141 samples were analyzed, consisting of 84 samples from 42 pairs of DNA-Reps, 74 samples from 37 pairs of Extract-Reps, and 62 samples from 21 trios of Ind-Reps (**Supplementary Table 1**). These accessions were originally collected from 12 countries (Brazil, Colombia, Cuba, Ecuador, Fiji, Guatemala, Mexico, Panama, Paraguay, Peru, Thailand, and USA). After evaluating these 21 accessions and their corresponding replicates, DNA extractions were performed on another 5,414 accessions from the cultivated cassava collection, originally from 28 different countries (**Table 1**). Among these accessions, 614 are declared as breeding lines while 4,800 are landraces. Including the 141 replicates, this study involved a total of 5,555 samples.

**Table 1:** Summary of the 5,414 accessions from the cultivated cassava collection used in this study.

Country of origin	Number of Breeders’ Lines	Number of Landraces	Total number of accessions	Number acc. In core	Region
ARG	6	105	111	5	Eastern South America
BOL		7	7	3	Eastern South America
BRA	86	1158	1244	93	Eastern South America
CHN		2	2	2	Asia
COL	439	1804	2243	155	Western South America
CRI		96	96	15	Central/North America & Caribbean
CUB	2	76	78	18	Central/North America & Caribbean
DOM		4	4	4	Central/North America & Caribbean
ECU		105	105	28	Western South America
FJI		5	5	1	Asia
GTM		78	78	10	Central/North America & Caribbean
HND		35	35	1	Central/North America & Caribbean
IDN	21	218	239	6	Asia
JAM		21	21	2	Central/North America & Caribbean
MEX	4	95	99	18	Central/North America & Caribbean
MYS	8	55	63	13	Asia
NGA	18		18	3	Africa
NIC		4	4		Central/North America & Caribbean
PAN		48	48	8	Central/North America & Caribbean
PER		395	395	72	Western South America
PHL	2	4	6	2	Asia
PRI		16	16	8	Central/North America & Caribbean
PRY	2	198	200	38	Eastern South America
SLV		11	11		Central/North America & Caribbean
THA	26	9	35	3	Asia
unknown		1	1		NA
USA		7	7	2	Central/North America & Caribbean
VEN		234	234	52	Western South America
VNM		9	9		Asia
Total	614	4,800	5,414	562	

### 2.2 DNA extractions and genotyping

DNA extractions were conducted between 2016 and 2021 as funding resources became available. Approximately 10 mg of lyophilized leaf tissue, obtained from *in-vitro* plantlets was used for DNA extraction on 96 well plates. Samples were homogenized at 12,000 rpm for 1 min using the Geno/Grinder 2010 (Spex SamplePrep LLC, NJ) and subsequently lysed with CTAB extraction buffer (containing 2M NaCl, 0.25M EDTA pH 8.0, 0.1M Tris-HCl pH 8.0, 2% CTAB, 2% PVP, and 0.2% 2-Mercaptoethanol) (Dellaporta et al., 1983). After mixing by vortex, samples were incubated at 65°C for 30 min, followed by the addition of an equal volume of chloroform:Isoamyl alcohol 24:1 (Sigma-Aldrich, USA). The resulting mixture was carefully mixed for 5 min and then centrifuged for 20 min at 3,000 rpm at 4°C. The aqueous phase was transferred to another tube and mixed with equal volume of cold isopropanol, followed by an incubation at -20°C for 1 h. After incubation, samples were centrifuged for 20 min at 3,000 rpm at 4°C. Upon removal of the supernatant, the pellets were washed with 80% cold ethanol and centrifuged again. Pellets were air-dried and resuspended in TE buffer (pH 8.0, Alpha Teknova, USA) with 40 µg of RNase (QIAGEN, Germany). The samples were then incubated at 37°C for 30 min and stored at -20°C. Samples were handled using multichannel pipettes. The concentration and purity of DNA was estimated by calculating the absorbance at 260/280 nm using Bio Teak Synergy H1m (Agilent Technologies, USA) and quality was assessed on a 0.8% agarose gel.

Fifty microliters of genomic DNA, with a concentration of 50 ng/µl for each sample, were submitted to Diversity Array Technology in Canberra, Australia, for genotyping by sequencing. DArTseqTM technology was employed, utilizing a combination of *MseI* and *PstI* restriction enzymes to prepare the genomic representation and subsequent next-generation sequencing as described by Kilian et al. (2012). Marker identification and allele-calling were performed with DS14 software (Diversity Arrays Technology P/L). In this study, two types of markers were utilized: codominant SNP markers and presence/absence dominant SilicoDArT markers.

### 2.3 Filters for high-quality marker and high-quality sample selection

A series of quality filters were carefully reviewed and applied in this study to select the most informative and high-quality markers, while excluding genomic representations prepared from low-quality DNA samples. For SNP markers, the selection process involved considering two parameters reflecting the markers’ information content, minor allele frequency (maf), estimated as the frequency at which the less-common allele of a genetic variant occurs within a population, and call rate, estimated as the proportion of samples for which the genotype is called and there is no missing value (Hernandez et al., 2019). Additionally, three parameters related to the technical aspects of marker quality were used, AvgMarkerCount, CVMarkerCount, and RepAvg. Average marker count (AvgMarkerCount) denotes the average number of sequence-tag copies of a marker, calculated by averaging the mean number of sequence-tag copies of the two SNP alleles. CVMarkerCount represents the coefficient of variation of AvgMarkerCount, utilized to minimize the chance of erroneously matched paralogue alleles from different loci. Additionally, RepAvg — an estimate calculated by DArT P/L — assessed the proportion of technical replicate assay pairs for which the calls of a given marker were consistent. A final filter was applied for markers that mapped to the cassava reference genome v7.1 (Bredeson et al., 2016). These filters were sequentially applied in the following order: maf ≥ 0.001, callrate ≥ 0.8, AvgMarkerCount ≥ 12, CVMarkerCount ≤ 0.6, RepAvg ≥ 0.98, and markers mapped to the reference genome v7.1.

For SilicoDArT dominant markers, a similar approach was used, with consideration given to two parameters reflecting the information content of markers call rate and OneRatio, and similar parameters related to the technical aspects of marker quality AvgReadDepth, CVReadDepth, and Reproducibility. OneRatio indicates the proportion of samples for which the genotype score was “1” (present). Average read depth (AvgReadDepth) denotes the average tag read count, calculated as the total sum of tag read counts across all samples divided by the number of samples’ score as “1”. CVReadDepth, which represents the coefficient of variation of AvgReadDepth, was utilized to remove markers with high variability in the number of tag read counts, potentially reflecting issues during PCR amplification. The filters were applied consecutively in the following order: call rate ≥ 0.95, OneRatio ≥ 0.05, AvgReadDepth ≥ 12, CVReadDepth ≤ 0.7, and Reproducibility ≥ 0.98. The impact of each filter on the number of markers and the genetic distances between pairs of replicates (DNA-Reps, Extract-Reps, and Ind-Reps) was thoroughly studied before settling on the selected values.

To eliminate low-quality samples that could potentially affect genetic-distance calculations, we assessed a range of parameters across 5,555 samples, including the 141 samples of the technical and biological replicates, and the 5,414 accessions from the cultivated cassava collection (**Table 1**). The parameters assessed included target quality control (QC), a categorical parameter provided by DArT P/L (Canberra, Australia),classifying library quality as “good”, “downshifted” or “weak”; total read count (tagcounttotal), unique read count (tagcountunique), individual SNP callrate, observed heterozygosity of individuals (Ho), individual SilicoDArT callrate, and individual SilicoDArT OneRatio, the latter represents the proportion of markers within one sample called as ‘1” (present). The total read count represents the total number of reads obtained per sample from sequencing each library. The three categories of target QC are evaluated on an agarose gel. A library categorized as ‘good’ exhibits DNA within the expected size range. ‘Downshifted’ libraries show DNA on a gel with a broader size range, shifting to smaller sizes, and may be associated with predigested DNA due to poor DNA quality. Libraries categorized as ‘weak’ are likely to have poor amplification due to uneven DNA concentrations. Each of these parameters was plotted to identify a suitable threshold for selecting high-quality samples, considering the target QC of samples. The thresholds used for each parameter to retain samples were tagcounttotal > 1,500,000, tagcountunique > 230,000, individual SNP callrate > 0.73, Ho > 0.05 and < 0.16, individual SilicoDArT callrate > 0.996 and, individual SilicoDArT OneRatio > 0.2. Subsequent analysis excluded samples failing to meet these criteria (**Supplementary Table 2**).

### 2.4 Calculation of genetic distances for SNP and SilicoDArT markers

For SNP markers, we calculated Identity-By-State (IBS) distances using the 1-IBS function in PLINK v1.0 (Purcell et al., 2007), released on June 29, 2007. The IBS calculation is based on counting alleles that are identical by state (IBS) between pairs of individuals at each genotyped marker. PLINK computes the IBS sharing proportion by comparing the number of shared alleles (IBS count) to the total non-missing alleles for each pair of individuals. This proportion is then subtracted from 1 to determine the distance between each pair of samples. IBS distances were independently calculated for several datasets. The first dataset comprised 141 samples, encompassing both technical and biological replicates from 21 accessions. IBS distances were calculated using these 141 samples and SNP marker sets before and after applying marker-quality filters, resulting in 22,840 to 7,001 SNPs (without excluding low-quality samples). The second dataset included a subset of 131 samples of technical and biological replicates, obtained after excluding 10 low-quality samples, and 6,987 SNP markers obtained after applying marker-quality filters. The third dataset consisted of 5,302 accessions, representing 95 percent of the cultivated cassava collection conserved at CIAT, and 7,180 SNP markers obtained after applying consecutive filters to the six marker quality parameters as described in section 2.3.

To calculate genetic distances for SilicoDArT markers, we used the gl.dist.ind function with the Jaccard method from the dartR package v2.0.4 (Gruber et al., 2018; Mijangos et al., 2022). The Jaccard distance matrix was calculated from the dataset of 131 samples, including technical and biological replicates, and 29,456 or 13,715 SilicoDArT markers obtained before and after marker-quality filters, respectively. Additionally, the Jaccard distance was calculated for 5,302 samples and 8,186 SilicoDArT markers after applying filters to marker quality parameters.

### 2.5 Genetic-distance threshold to identify genetically redundant accessions

To identify genetic distinctness and redundancy within the cassava collection, we initially estimated the average genetic distances among the three types of pairs of replicates (DNA-Reps, Extract-Reps, and Ind-Reps) from 21 accessions from the core collection. This step aimed to evaluate the efficacy of selected marker-quality filters in retaining a high number of markers while achieving minimal (close to zero) genetic-distance estimates for replicate pairs. To assess the threshold’s ability to detect genetically redundant accessions, we utilized the mlg.filter function from the poppr package v2.9.3 (Kamvar et al., 2014), in R program v4.2.2 (R Core Team, 2022), following the methodology outlined by Albuquerque et al. (2019). This function collapses multilocus genotypes (MLGs) falling below a specific threshold based on genetic distance.

In our analysis using the SNP dataset, we conducted two tests from the 21 subset accessions: one test, comprised a dataset with 141 samples and 7,001 SNP markers. We empirically set the minimum genetic distance at 0.06, informed by the maximum observed IBS distance among replicates. A second test, with the 21 accessions, excluded 10 low-quality samples, resulting in a dataset of 131 samples and 6,987 SNP markers. Here, the minimum genetic distance was set at 0.015, determined from new estimations of average genetic distance between pairs of replicates after removing the 10 low-quality samples (**Supplementary Figure 2**). Subsequently, we performed the final analysis to detect the number of MLGs using the IBS distance matrix calculated across 5,302 accessions from the cultivated cassava collection and 7,180 SNP markers. Additionally, we explored the impact of varying the threshold for cluster identification, considering minimum IBS thresholds ranging from 0.000 to 0.06.

In parallel, we detected MLG using SilicoDArT markers and Jaccard distance, using the 131 samples and 13,715 SilicoDArT markers, exploring different thresholds (0.012 and 0.025). Furthermore, we detected MLG using the Jaccard distance, estimated for the larger dataset comprising 5,302 accessions and 8,186 SilicoDArT markers.

### 2.6 Clustering analyses

We performed agglomerative clustering using the complete linkage method to assess and validate the MLGs identified across the 141 and 131 samples. Additionally, we conducted agglomerative clustering employing the ward.D2 linkage method with the 5,302 accessions to discern potential patterns of genetic redundancy within/across regions of origin, and to identify discrepancies between the results obtained with the two marker types (Murtagh and Legendre, 2014). The clustering algorithm utilized either IBS or Jaccard genetic distances and was implemented through the hclust function in the stats R package v4.2.2 (R Core Team, 2022). The resulting hierarchical clusters were visualized directly or transformed into Newick trees using the cluster-to-tree conversion function hc2Newick from the ctc R package v1.72.0. The Newick trees were customized and annotated using the Interactive Tree of Life (iTOL) online tool (Letunic and Bork, 2021).

### 2.7 Comparing and contrasting datasets and data types

To compare the results obtained from the MLG detection analysis using the two types of genetic markers and the derived genetic distances (Jaccard and IBS distances), we generated Venn diagrams to compare the accessions detected as unique or as redundant within the MLGs detected. For this purpose, we utilized the venn.diagram function from the VennDiagram R package v1.7.3.

Additionally, a subset of 20 MLGs of varying sizes (collapsing 2, 3, 4, 6, 7, 12, 28, 29, 31, and 84 accessions) were used to review passport and historical characterization data with clusters. The reviewed passport data included: biological status (landrace or breeding germplasm), country and region of origin, common names, and collection date. The historical characterization variables included color root pulp, shape of central leaf, number of leaf lobes, petiole color, and color of the first expanded leaf. The historical characterization data and images were extracted from Genesys, 2023 (https://www.genesys-pgr.org/datasets/; https://www.genesys-pgr.org/a/images/).

## 3 Results

### 3.1 Defining empirical thresholds for detecting genetically redundant accessions

**Figure 1:**
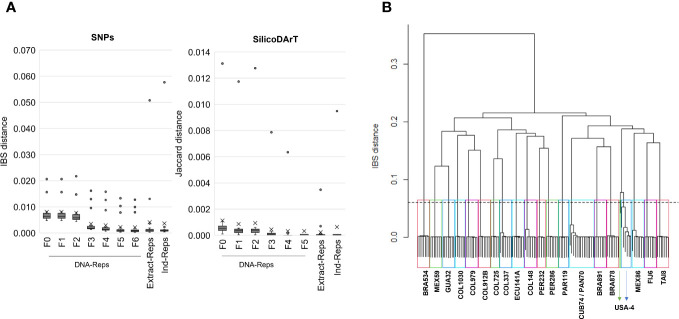
**(A)** The cumulative impact of implementing exemplary thresholds for each marker-quality parameter on genetic distances. The box plots display the distribution of the genetic distance of technical and biological replicates at various stages of filter application, from F0 (unfiltered data) to F6. F0 to F6 indicate distances of DNA-Rep pairs across filtering steps. Extract-Rep and Ind-Rep pairs distances were estimated with filters in F6 for SNP or F5 for SilicoDArT. **(B)** Dendrogram of hierarchical clustering using the “complete linkage” method and the IBS distance. Twenty-one rectangles highlight the 21 clusters formed under a genetic distance of 0.06 (dashed line). Accession names representing samples within each group are shown. The IBS distance matrix was calculated from 141 samples and 7,001 SNP markers.

A total of 22,840 polymorphic SNPs were detected across 141 samples from 21 cassava accessions selected from the core collection, including their corresponding biological and technical replicates. The application of six consecutive filters to the marker-quality parameters reduced the number of markers from 22,840 to 7,001 SNPs. The estimated average genetic distance for the 42 DNA-Reps pairs decreased from 0.008 (± 0.004 SD) to 0.002 (± 0.003 SD) (see F0 to F6 in **Table 2**; **Figure 1A**). The average genetic distance of the 37 Extract-Rep and 21 Ind-Rep pairs remained relatively higher (0.004 ± 0.011 and 0.003 ± 0.012, respectively; **Table 2**; **Figure 1A**). Because the highest genetic distance value for Ind-Reps was 0.0576, the initial minimum distance to distinguish genetically unique accessions within this validation set of 141 samples was set at 0.06. The MLG analysis detected 21 different MLGs, representing 18 genetically unique accessions (each with their respective technical and biological replicates), one group of two genetically redundant accessions with their technical and biological replicates (CUB74, PAN70; MLG 10, **Table 3**), and two groups with replicates of accession USA4 (MLGs 20 and 21, **Table 3**; **Figure 1B**). The division of USA4 samples into two distinct clusters points to potential issues with DNA sample quality.

Table 2Combining multiple marker-quality thresholds.

**Table:** 

Order	Parameter used SNPs	DNA-Reps							Extract-Reps	Ind-Reps
		F0	F1	F2	F3	F4	F5	F6	F6	F6
**1**	**maf**		≥ 0.001	≥ 0.001	≥ 0.001	≥ 0.001	≥ 0.001	≥ 0.001	≥ 0.001	≥ 0.001
**2**	**Callrate Loc**			≥0.8	≥0.8	≥0.8	≥0.8	≥0.8	≥0.8	≥0.8
**3**	**AvgMarkerCount**				≥12	≥12	≥12	≥12	≥12	≥12
**4**	**CVMarkerCount**					≤0.6	≤0.6	≤0.6	≤0.6	≤0.6
**5**	**RepAvg**						≥ 0.98	≥ 0.98	≥ 0.98	≥ 0.98
**6**	**Mapped genome v7**							yes	yes	yes
**SNP**	**Number of SNPs**	22,840	22,840	17,221	8,768	8,400	7,717	7,001	7,001	7,001
	**mean dist**	0.008	0.008	0.0077	0.0035	0.0029	0.0022	0.0022	0.0041	0.0037
	**SD dist**	0.0048	0.0048	0.0053	0.0041	0.0041	0.0036	0.0036	0.0113	0.0127
	**max. dist**	0.0212	0.0212	0.0043	0.0161	0.0157	0.0132	0.0127	0.0507	0.0576
	**min. dist**	0.0047	0.0047	0.021	0.0013	0.0006	0.0003	0.0002	0.0002	0.0004

**Table:** 

Order	Parameter* used	DNA-Reps							Extract-Reps	Ind-Reps
		F0	F1	F2	F3	F4	F5		F5	F5
**1**	**Callrate Loc**		≥ 0.95	≥ 0.95	≥ 0.95	≥ 0.95	≥ 0.95		≥ 0.95	≥ 0.95
**2**	**OneRatio**			≥0.05	≥0.05	≥0.05	≥0.05		≥0.05	≥0.05
**3**	**AvgReadDepth**				≥12	≥12	≥12		≥12	≥12
**4**	**CVReadDepth**					≤0.7	≤0.7		≤0.7	≤0.7
**5**	**Reproducibility**						≥ 0.98		≥ 0.98	≥ 0.98
SilicoDArT	**Number of SilicoDArT**	29,456	26,015	23,674	16,664	14,089	13,715		13,715	13,715
	**mean dist**	0.0011	0.0008	0.0009	0.0004	0.0003	0.0003		0.0002	0.0006
	**SD dist**	0.0027	0.0024	0.0027	0.0017	0.0016	0.0014		0.0007	0.0022
	**max. dist**	0.0131	0.0117	0.0127	0.0078	0.0063	0.0063		0.0034	0.0094
	**min. dist**	0.0001	0.0000	0.0000	0.0000	0	0		0	0.0000

*Minor allele frequency (maf), frequency at which the less-common allele of a genetic variant occurs within a population; Call rate, estimated as the proportion of samples for which the genotype is call and there is no missing value; AvgMarkerCount, the average number of sequence-tag copies of a marker; CVMarkerCount, the coefficient of variation of AvgMarkerCount; RepAvg, the proportion of technical-replicate assay pairs for which the calls of a given marker were consistent; OneRatio, the proportion of samples for which the genotype score was “1” (present); AvgReadDepth, the average tag read count, calculated as the total sum of tag read counts across all samples divided by the number of samples score as “1”; CVReadDepth, the coefficient of variation of AvgReadDepth.

List of parameters for SNP and SilicoDArT markers, order of application, starting from no filters applied (F0) to all filters applied (F6). Number of markers, the mean of genetic distance (IBS for SNPs and Jaccard for SilicoDArT), standard deviation (SD) of pairs of DNA, Extract-Reps, and Ind-Reps.

**Table 3:** Summary of multilocus genotype (MLG) detection using SNP and SilicoDArT markers in 21 accessions, including biological and technical replicates.

Accessions	SNP−IBS distance				SilicoDArT−Jaccard distance					
	Threshold 0.06*		Threshold 0.015**		Threshold 0.012**		Threshold 0.020**		Threshold 0.025**	
	MLG size	MLG ID	MLG size	MLG ID	MLG size	MLG ID	MLG size	MLG ID	MLG size	MLG ID
BRA534	7	1	7	1	7	1	7	1	7	1
BRA878	7	2	7	2	7	2	7	2	7	2
BRA891	7	3	7	3	7	3	7	3	7	3
COL1030	6	4	5	4	5	4	5	4	5	4
COL148	7	5	5	5	5	5	5	5	5	5
COL337	7	6	5	6	5	6	5	6	5	6
COL725	5	7	5	7	5	7	5	7	5	7
COL912B	7	8	7	8	7	8	7	8	7	8
COL979	7	9	7	9	7	9	7	9	7	9
CUB74/PAN70	14	10	13	10	13	10	13	10	13	10
ECU141A	7	11	7	11	7	11	7	11	7	11
FJI6	7	12	6	12	6	12	6	12	6	12
GUA32	7	13	7	13	7	13	7	13	7	13
MEX59	7	14	7	14	7	14	7	14	7	14
MEX86	7	15	7	15	1	15	1	15	7	15
PAR119	6	16	6	16	6	16	6	16	6	16
PER232	5	17	5	17	5	17	5	17	5	17
PER286	7	18	6	18	6	18	6	18	6	18
TAI8	7	19	7	19	7	19	7	19	7	19
USA4	1*	20	5	20	1	20	5	20	5	20
USA4	6*	21	–	–	1	21	–	–	–	–
USA4	–	–	–	–	3	22	–	–	–	–
MEX86	–	–	–	–	6	23	6	21	–	–
TOTAL	141	21	131	20	131	23	131	21	131	20

*dataset with 141 samples; **dataset with 131 samples.

Minimum genetic distance thresholds are specified along with MLG size and MLG number. Accessions with (**+**) indicate multiple MLG detection for at least one marker or distance threshold.

### 3.2 Sample quality assessment

**Figure 2:**
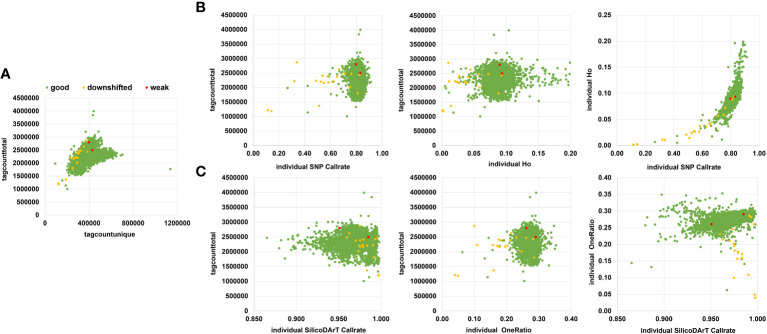
Sample quality parameters were assessed to filter out low-quality samples. **(A)** Scatter plot displaying tagtotalcounts vs. taguniquecounts per sample. **(B)** Scatter plot showing the relationship between tagtotalcount vs individual SNP call rate or individual observed heterozygosity (Ho), or individual call rate vs. Individual Ho. **(C)** Scatter plot using SilicoDArTs, illustrating the relationship between tagtotalcount vs. individual call rate or individual oneRatio. Furthermore, it includes a comparison of call rate vs. oneRatio for SilicoDArT markers. Samples are color-coded according to their target quality-control assessment on a gel, categorized as good (green), downshifted (yellow), or weak (red).

We assessed seven different quality parameters across 5,555 samples from the cultivated cassava collection, which included the 5,414 accessions described in **Table 1**, as well as 21 accessions along with their 141 technical and biological replicates (**Supplementary Table 1**). These parameters included target QC, tagcounttotal, tagcountunique, individual SNP call rate, Ho of individuals, individual SilicoDArT call rate, and individual SilicoDArT OneRation. Among the 5,555 samples, 5,532 targets (commonly known as sequenced DNA libraries) were categorized as “good”, 21 were classified as “downshifted”, and two as “weak”. The assessment of target QC was conducted by DArT P/L and was based on agarose gel evaluation (specific data not provided). The statistical distribution of other associated sample quality parameters revealed that the mean values of tagcounttotal and tagcountunique were 2,306,247 (ranging from 1,007,722 to 3,995,430) and 385,875 (ranging from 92,395 to 1,136,722) reads, respectively. Seven samples exhibited remarkably low levels of tagcounttotal, falling below 1,500,000 read counts, while 27 samples displayed tagcountunique values lower than 230,000 (**Supplementary Table 2**). Regarding sample quality of SNP-related parameters, such as call rate and Ho, the mean values were 0.83 (ranging from 0.11 to 0.91) and 0.09 (ranging from 0 to 0.2), respectively. Notably, most of the downshifted samples had call rates below 0.73 (a total of 72 samples), and/or Ho values either below 0.05 or above 0.16 (79 samples) (**Supplementary Table 2**; **Figure 2**).

The sample’s SilicoDArT-related parameters, including call rate and OneRatio, had mean values of 0.97 (ranging from 0.86 to 1) and 0.27 (ranging from 0.04 to 0.35), respectively. Notably, 30 samples had a call rate above 0.996, and 21 samples exhibited OneRatio values below 0.2. By visually representing these parameters and employing distinct colors for the three target QC categories, we observed that a majority of the downshifted samples (18 samples) fell below or above the designated thresholds (**Figure 2**; **Supplementary Table 2**). In total, 101 samples fell below or above the threshold for at least one of these parameters, while 42 samples exhibited disparities in 2 to 6 parameters (**Supplementary Table 3**). A total of 143 samples out of the initial 5,555 were excluded from further analysis as they were identified with low-quality. Out of these 143, ten samples were drawn from the set of 141 biological and technical replicates (**Supplementary Table 3**). This resulted in a dataset comprising 131 samples and 6,987 SNP markers, which was used for establishing the genetic distance threshold and validating the detection of MLGs. Consequently, after removing low-quality samples a total of 5,302 accessions from the cultivated cassava genebank collection were retained for the MLG detection analysis.

### 3.3 Verification of minimum genetic distances for MLG detection after removing low-quality samples

Comparing the genetic distances among the dataset of 141 or 131 technical and biological replicates (before and after filtering samples), showed a significant reduction in the genetic distance variation within replicates of four accessions, COL148, CUB74, MEX86, and notably in the case of USA4 (**Supplementary Figure 2**). Consequently, this reduction in the genetic distance after removing low quality samples allowed us to set the minimum genetic distance for MLG detection to 0.015. Using this threshold and the dataset of 131 samples, we repeated the MLG detection procedure. This process validated the accuracy of the approach to identify groups of distinct genotypes for the replicates derived from each accession. The analysis identified 20 MLGs, as documented in **Table 3**, collapsing all replicates from each accession into distinct MLGs, including replicates from USA4. As previously observed, two accessions (CUB74 and PAN70) were identified within the same MLG.

Moreover, we assessed the number of MLGs by calculating Jaccard genetic distance from the SilicoDArT markers to the selected 131 high-quality samples. The initial number of SilicoDArT markers for this set of samples was 29,456. Similarly, as with the SNPs, a series of marker quality parameters were assessed including call rate, OneRatio, AvgMarkerCount, CVMarkerCount and Reproducibility. Applying filters to these parameters reduced markers from 29,456 to 13,715 as shown in **Table 2**. The estimated average genetic distance of the 42 pairs of DNA-Reps decreased from 0.0011 ± 0.002 to 0.0003 ± 0.0014 (see F0 to F5 in **Table 2**; **Figure 1A**). Moreover, the mean genetic distance for the 37 Extract-Rep and 21 Ind-Rep pairs measured 0.0002 ± 0.0007 and 0.0006 ± 0.0022, respectively (**Table 2**; **Figure 1A**). When utilizing a minimum Jaccard genetic distance threshold of 0.012 for MLG detection, a total of 23 unique MLGs were detected. Notably, two of these MLGs (15 and 23) corresponded to independent MLGs, including separately technical replicates from the MEX86 accession. Similarly, another three MLGs (20, 21, and 22) were identified for different technical replicates of the USA-4 accession (**Table 3**). These results suggested that a higher threshold for Jaccard distances needs to be used to allow all replicates from each accession to collapse in the same MLGs.

**Figure 3:**
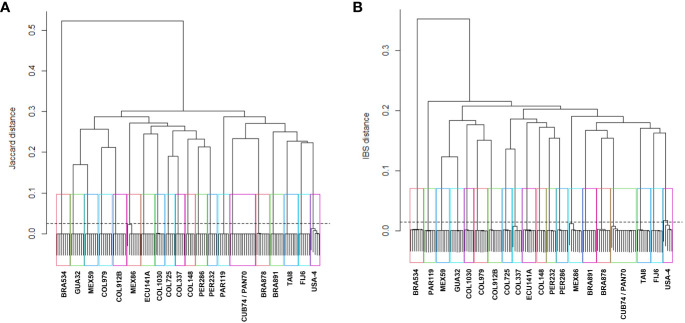
Dendrogram of hierarchical clustering using the “complete” method with the IBS distance for SNPs **(A)**, and Jaccard distance for SilicoDArTs **(B)**. Twenty rectangles highlight the 20 clusters formed under a genetic distance of 0.015 or 0.025 using SNP and SilicoDArT (dashed line) markers, respectively. Accession names representing samples within each group are shown. The distance matrices were calculated from 131 samples and 6,987 SNP or 13,715 SilicoDArT markers.

By increasing the minimum Jaccard genetic distance to 0.025, samples were collapsed into 20 MLGs, similarly to the SNP markers when using a minimum IBS genetic distance threshold of 0.015 (**Table 3**). The hierarchical clustering analysis showed 20 clusters below a threshold of 0.015 for IBS and 0.025 Jaccard distances (**Figure 3**). Notably, all technical replicates from each accession clustered together, including those from MEX86 and USA4. Consistently, two accessions were grouped together (CUB74/PAN70), highlighting an instance of redundant accessions within the dataset.

### 3.4 Assessing genetic redundancy within the cultivated cassava genebank collection

**Figure 4:**
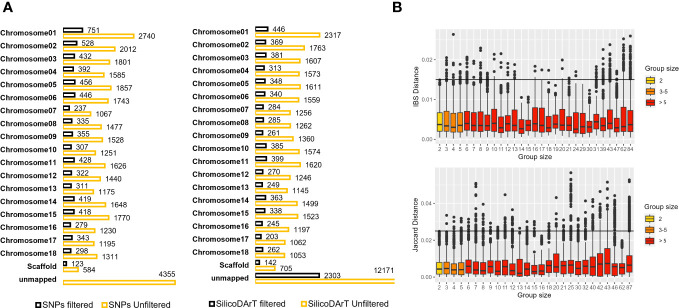
**(A)** Total SNP and SilicoDArT markers mapped or unmapped to the cassava reference genome v7 before and after applying filters (see **Supplementary Table 4**). **(B)** Dispersion of genetic distances among pairs of accessions within multi-accession MLGs of varying group sizes. The upper boxplot displays IBS distances estimated from SNPs, with MLG group sizes ranging from 2 to 84. The lower boxplot shows Jaccard distances estimated from SilicoDArT markers, with MLG group sizes ranging from 2 to 87. Group sizes are categorized and represented in different colors. Thresholds 0.015 and 0.025 are shown with a black line.

We utilized a validated approach, carefully selecting high-quality samples and markers. We also established a minimum genetic distance threshold for IBS and Jaccard distances, allowing us to distinguish distinct and redundant accessions. With this approach, we evaluated genetic redundancy in 5,302 accessions, covering 95 percent of CIAT’s cultivated cassava collection and 88 percent of the entire collection, as shown in **Table 1**. Initially, we had 33,395 polymorphic SNPs and 39,103 SilicoDArTs. After subjecting these markers to quality assessments (refer to **Supplementary Figure 3**) and applying the same filtering thresholds as for the replicates’ dataset, we ended up with a refined set of 7,180 SNP and 8,186 SilicoDArT markers (see **Supplementary Table 4**). These markers were distributed across the 18 chromosomes of the cassava reference genome, maintaining proportional representation across chromosomes before and after filtering markers, as illustrated in **Figure 4A**. Initially, 29,040 unfiltered SNPs were mapped across the reference genome, while 4,355 remained unmapped. Following the application of filters, we obtained 7,180 SNPs mapped to the reference genome. In the case of SilicoDArTs, 26,932 unfiltered markers were mapped, leaving 12,171 unmapped. In the final filtering process, 5,883 SilicoDArT markers were mapped and 2,303 remained unmapped.

The MLG detection analysis, using the IBS genetic-distance matrix, identified a total of 1,567 distinct genotypes, each represented by only one accession per MLG (clusters with one accession or C1), and hereafter referred to as “single-accession MLGs”. A total of 3,735 accessions were collapsed within 951 MLGs, ranging from 2 to up to 84 accessions per cluster (denoted in **Table 4** as C2 to C84 in the MLG size column) and hereafter referred to as “multi-accession MLGs”. In total, the analysis using SNPs identified 2,518 unique MLGs across the 5,302 accessions (**Table 4**) and 2,784 accessions were detected as redundant. The MLG detection analysis using the Jaccard genetic-distance matrix identified a total of 1,568 single-accession MLGs. A total of 3,734 accessions were collapsed within 958 multi-accession MLGs containing from 2 to 87 accessions (C2 to C87). SilicoDArT marker analysis identified a total of 2,526 unique MLGs across the 5,302 accessions, and 2,776 accessions were detected as redundant. The two genetic distance measures showed that 47 percent of the genotypes were distinct or unique (47.5 percent and 47.6 percent for SNPs and SilicoDArT, respectively), while 52 percent were redundant (**Table 4**). Furthermore, we examined the dispersion of genetic distances among accessions collapsed within “multi-accession MLGs” of varying sizes, ranging from 2 to 84/87 accessions (as indicated in **Table 4**, MLG size C2 to C87). Interestingly, pairs of accessions within these MLGs exhibited dispersion of genetic distances above the given threshold for MLG detection (**Figure 4B**).

**Table 4:** Summary of MLG detection with SNP and SilicoDArT markers across 5,302 accessions using genetic distances thresholds of 0.015 and 0.025 for IBS and Jaccard distances, respectively.

MLG size	SNP		SilicoDArT		Color code
	Number of MLGs	Number of acc. in MLGs	Number of MLGs	Number of acc. in MLGs	
C1	1567	1567	1568	1568	Green
C2	472	944	495	990	Yellow
C3	191	573	174	522	Orange
C4	96	384	101	404	
C5	61	305	56	280	
C6	31	186	32	192	Red
C7	21	147	21	147	
C8	14	112	14	112	
C9	15	135	14	126	
C10	8	80	9	90	
C11	8	88	9	99	
C12	6	72	7	84	
C13	7	91	3	39	
C14	2	28	4	56	
C15	1	15	3	45	
C16	3	48	2	32	
C17	1	17	–	–	
C18	1	18	1	18	
C19	1	19	–	–	
C20	1	20	2	40	
C21	1	21	1	21	
C24	1	24	–	–	
C25	–	–	1	25	
C29	1	29	–	–	
C30	1	30	2	60	
C31	1	31	–	–	
C32	–	–	1	32	
C39	1	39	–	–	
C40	–	–	1	40	
C42	–	–	1	42	
C43	2	86	1	43	
C46	–	–	1	46	
C47	1	47	–	–	
C62	1	62	1	62	
C84	1	84	–	–	
C87	–	–	1	87	
Total	2518	5302	2526	5302	
Num. redundant	2784		2776		
% of distinct	47.49		47.64		
% of redundant	52.51		52.36		

**Figure 5:**
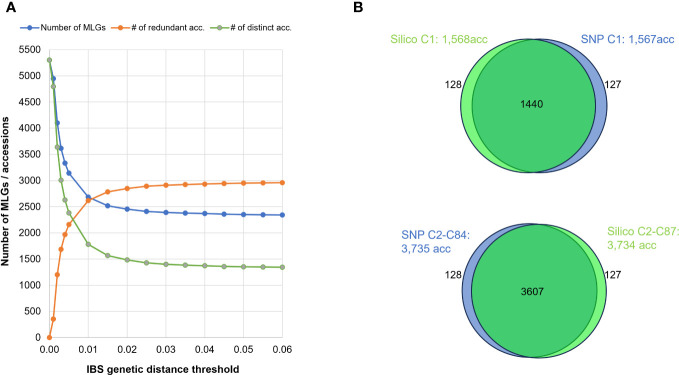
**(A)** Effect of varying IBS distance thresholds on MLG detection: Blue line represents the number of MLGs detected when applying different minimum genetic-distance thresholds. Green line represents the number of distinct accessions. Orange line indicates the number of genetically redundant accessions. **(B)** Venn diagrams illustrating intersection of accessions detected by SNP (in blue) or SilicoDArT (in green) markers in MLGs, differentiating between single-accession MLGs (Upper) and multi-accession MLGs (Lower).

Additionally, we investigated the effect of varying the threshold for MLG identification using the SNP dataset, by running a series of MLG analyses and using IBS distance thresholds ranging from 0.000 to 0.06. When using thresholds between 0.01 and 0.06, the number of clusters varied moderately between 2,648 and 2,343. However, for thresholds below 0.01, the number of clusters increased substantially to more than 5,000 MLGs, most likely because of genotyping errors artificially inflating genetic distances (**Figure 5A**).

### 3.5 Comparative analysis of genetic redundancy assessment approaches

When comparing the various MLGs detected among the 5,302 accessions using both IBS and Jaccard distances, both approaches (SNP and SilicoDArT, respectively) consistently identified 5,047 accessions as either distinct or redundant. The remaining 255 accessions were not detected by either method. Among the 5,302 accessions, 1,440 were commonly detected within single-accession MLGs (datasets: “Silico C1: 1,568acc” and “SNP C1: 1,567acc”), while 3,607 accessions were identified within multi-accession MLGs (datasets: “Silico C2-C87: 3,734acc”, “SNP C2-C84: 3,735acc”) (**Table 4**; **Figure 5B**). Within the group of accessions detected as redundant by both approaches, 119 accessions were detected in different MLG sizes by each method (SNPs and SilicoDArT). Additionally, 128 accessions were detected as single-accession MLGs with SilicoDArT markers, but those accessions were detected within multi-accession MLGs by SNPs. Conversely, 127 accessions were detected as single-accession MLGs by SNPs but were detected within multi-accession MLGs by SilicoDArTs (**Figure 5B**). A total of 374 accessions showed discrepancies in the detection of MLGs by both types of markers (119 + 127 + 128 = 374).

We classified MLGs into four distinct groups based on their sizes: single-accession MLGs (C1) coded in green, two-accession MLGs (C2) coded in yellow, and two additional categories for multi-accession MLGs collapsing over two accessions. The first group comprised MLGs with 3−5 accessions (C3−C5) coded in orange, while the second group included MLGs with more than five accessions (C6 to C87) coded in red (refer to the last column in **Table 4**). Furthermore, we categorized accessions into five regions based on their countries of origin: western South America, eastern South America, Central/North America & Caribbean, Asia, and Africa (see **Table 1**). We then evaluated the number and percentage of accessions per region falling within the four distinct groups of MLG sizes (**Table 5**; **Supplementary Figure 4**). The most represented region within the collection is western South America (2,957 accessions) composed by Colombia, Venezuela, Ecuador, and Peru; followed by eastern South America (1,511 accessions) composed by Brazil, Paraguay, Bolivia and Argentina. Among the five regions, Africa and Asia showed the highest percentages of distinctness, meaning that they are composed mostly of single-accession MLGs, with unique genotypes recorded for 68 and 39 percent of the accessions within each region, respectively. Central/North America & Caribbean, eastern South America and western South America were the regions showing the highest percentages of redundancy, with 83,73 and 68 percent, respectively (**Table 5**). On the other hand, western South America and eastern South America had the highest values of discrepancies across the results obtained from the two types of markers (SNP and SilicoDArT).

**Table 5:** Distribution of accessions categorized by region of origin and four distinct MLG size categories: single-accession MLGs (C1), two-accession MLGs (C2), MLGs with 3 to 5 accessions (C3−C5), and MLGs with more than 5 accessions (C6−C84/C87).

Type of marker	Regions		Western South America	Eastern South America	Central/North America & Caribbean	Asia	Africa	Total
	Parameter	Total	2967	1511	451	354	19	5302
SNP	Number of accessions	C1	943	399	74	138	13	1567
		C2	523	287	67	61	6	944
		C3-C5	606	413	108	81		1208
		C6-C84	841	412	202	74		1529
	Percentage	distinctness	31.8	26.4	16.4	39.0	68.4	
		redundancy	68.2	73.6	83.6	61.0	31.6	
SilicoDArT	Number of accessions	C1	947	400	70	138	13	1568
		C2	546	301	71	66	6	990
		C3-C5	624	400	104	78		1206
		C6-C87	850	410	206	72		1538
	Percentage	distinctness	31.9	26.5	15.5	39.0	68.4	
		redundancy	68.1	73.5	84.5	61.0	31.6	
Both	Number of accessions	Discrepancy	214	114	30	16	0	374

The hierarchical clustering analysis of the 5,302 accessions, using IBS and Jaccard distances, revealed the presence of at least three major groups (**Supplementary Figure 4**). The first major group predominantly comprises accessions from western South America and Central/North America & the Caribbean, a second group encompasses a blend of accessions from eastern South America, western South America, Asia, and Africa. The third group predominantly comprises accessions from eastern South America (**Table 1**; **Supplementary Figure 4**). Both types of markers produced similar trees with slight differences. Although the purpose of this study is not to delve deeply into the population structure of the collection, the dendrogram helps visualize how single-accession MLGs and multi-accession MLGs are widely spread across the three major groups, with some differences across regions, as shown in **Table 5**. The discrepancies across both approaches (SNP and SilicoDArT) are also widely spread across the three clusters (**Supplementary Figure 4**).

### 3.6 Examining passport and historical data in genetically redundant accessions

Twenty cases of multi-accession MLGs with varying sizes, ranging from 2 to 84, were selected to review the passport and historical characterization data. The biological status, country of origin, common names and collection dates were reviewed from the passport data. The analysis revealed that 10 cases shared the same biological status of Landrace or Breeding Line (refer to counts of ones across cases in **Table 6**), while another 10 cases have discrepancies. On the other hand, 10 cases included accessions from multiple countries ranging from 2 to 6, while the other 10 cases included accessions from only one country of origin (cases 1, 2, 3, 5, 7, 9, 10, 11, 12, and 17). Only one case among the 20 had accessions with identical common names within a single MLG (case 9). The remaining cases consist of MLGs with accessions known with multiple names ranging from 2 to 52 different names (**Table 6**). Regarding the historical characterization data, five descriptors were reviewed, including shape of central leaf, petiole color, color of the first expanded leaf, number of leaf lobes and color of root pulp. Data were not available for all accessions. The percentage of accessions with available information for each descriptor varied from 20 percent for the number of leaf lobes, to 21 percent for color of the first expanded leaf, to 22 percent for petiole color, to 33 percent for shape of the central leaf, and to 77 percent for the color of root pulp (**Table 6**; **Supplementary Table 5**). We evaluated the variations within each MLG by assigning a value of 1 when all accessions shared the same descriptor and 2 or above when descriptors were different for at least one accession within an MLG. Additionally, we included the number of accessions with information for that particular descriptor, considering the total number of accessions within each MLG (**Table 6**).

**Table 6:** Summary of passport and historical characterization data for selected multi-accession MLGs.

Cases		1	2	3	4	5	6	7	8	9	10	11	12	13	14	15	16	17	18	19	20	Counts of ones
MLG	Size	2	2	2	2	2	2	2	2	2	3	3	4	6	7	7	12	28	29	31	84	
	Name (SNP/SilicoDArT)	4/65	5/5	13/2977	25/2472	37/37	42/42	47/47	49/49	207/207	11/5157	35/4918	16/4775	2468 /3121	393/164	2525/2525	1168/1595	1182/4747	2313/2459	739/1273	4535/4313	
Passport Data	BiologicalStatus	1 (2/2)	1 (2/2)	1 (2/2)	2 (2/2)	2 (2/2)	2 (2/2)	1 (2/2)	2 (2/2)	1 (2/2)	1 (3/3)	1 (3/3)	1 (4/4)	2 (6/6)	2 (7/7)	1 (7/7)	1 (12/12)	2 (28/28)	2 (29/29)	2 (31/31)	2 (84/84)	10
	CountriesOrigin	1 (2/2)	1 (2/2)	1 (2/2)	2 (2/2)	1 (2/2)	2 (2/2)	1 (2/2)	2 (2/2)	1 (2/2)	1 (3/3)	1 (3/3)	1 (4/4)	5 (6/6)	2 (7/7)	3 (7/7)	3 (12/12)	1 (28/28)	6 (29/29)	5 (31/31)	5 (84/84)	10
	CommonNames	2 (2/2)	2 (2/2)	ND	2 (2/2)	ND	2 (2/2)	2 (2/2)	2 (2/2)	1 (2/2)	3 (3/3)	ND	2 (2/4)	5 (5/6)	6 (7/7)	5 (5/7)	10 (12/12)	11 (24/28)	18 (25/29)	24 (31/31)	52 (78/84)	1
	CollectionDates	ND	2	ND	ND	2	ND	ND	ND	2	1	ND	1	2	4		1	21	13	11	54	
Characterization Data	ShapeCentralLeaf	2 (2/2)	1 (2/2)	2 (2/2)	1 (1/2)	2 (2/2)	1 (2/2)	1 (2/2)	1 (2/2)	2 (2/2)	2 (3/3)	2 (3/3)	3 (4/4)	2 (6/6)	2 (6/7)	3 (7/7)	4 (10/12)	3 (26/28)	5 (27/29)	4 (18/31)	4 (77/84)	4
	PetioleColor	1 (2/2)	1 (1/2)	1 (1/2)	ND	ND	ND	1 (1/2)	1 (2/2)	1 (2/2)	ND	2 (3/3)	2 (4/4)	2 (3/6)	2 (3/7)	1 (1/7)	4 (8/12)	2 (3/28)	3 (7/29)	3 (7/31)	3 (4/84)	3
	Color1st ExpandedLeaf	1 (2/2)	1 (1/2)	1 (1/2)	ND	ND	ND	1 (1/2)	1 (2/2)	1 (2/2)	ND	1 (3/3)	2 (4/4)	1 (3/6)	1 (3/7)	1 (1/7)	2 (7/12)	1 (3/28)	1 (7/29)	1 (7/31)	2 (4/84)	10
	NumberLeafLobes	2 (2/2)	1 (1/2)	1 (1/2)	ND	ND	ND	1 (1/2)	2 (2/2)	1 (2/2)	ND	1 (3/3)	1 (4/4)	1 (3/6)	2 (3/7)	1 (1/7)	1 (7/12)	1 (3/28)	1 (5/29)	1 (5/31)	2 (4/84)	9
	ColorRootPulp	1 (2/2)	1 (2/2)	1 (2/2)	1 (1/2)	1 (2/2)	1 (2/2)	1 (2/2)	ND	1 (2/2)	1 (3/3)	2 (3/3)	2 (4/4)	1 (5/6)	3 (6/7)	1 (7/7)	1 (7/12)	1 (25/28)	1 (26/29)	1 (11/31)	3 (77/84)	14
	Counts of ones	5	7	6	2	2	2	7	3	7	4	4	4	3	1	2	4	4	3	3	0	
	Counts ND	1	0	2	4	4	3	1	2	0	3	2	0	0	0	0	0	0	0	0	0	

The name row indicates the ID of the MLG with the SNP and SilicoDArT analyses, respectively. The first number in the body of the table indicates the number of classes found within a given MLG and passport or characterization variable. The numbers in parentheses indicate the number of accessions with data available. ND indicates that data was not available. Cases and variables where only one category across the accessions included are summarized in the “counts of ones” row and column.

**Figure 6:**
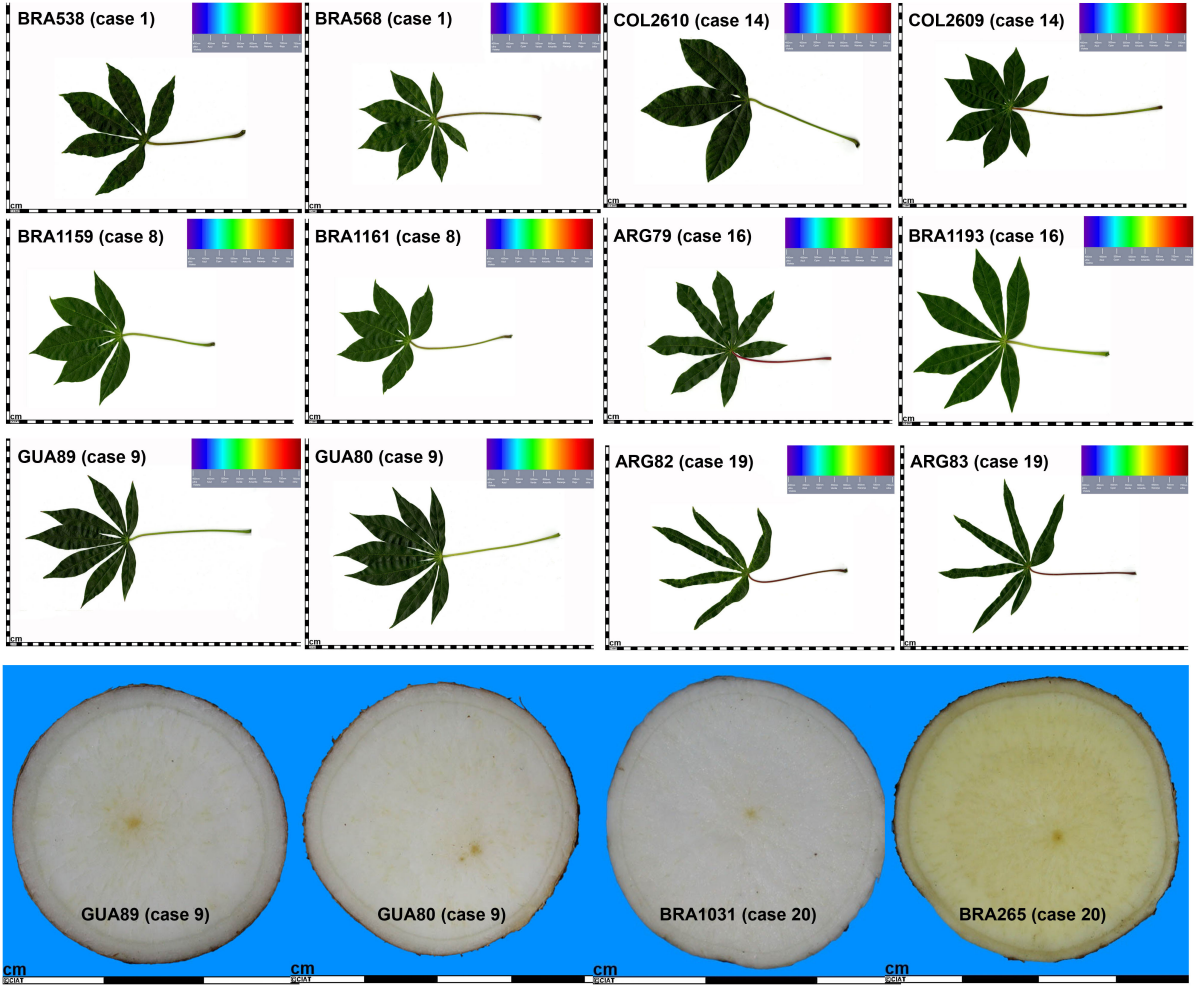
Selected cases of multi-accession MLGs, displaying historical leaf (top) and root (bottom) images from accessions within varied-sized MLGs, contrasting similarities, or differences in shape of central leaf, petiole color, number of leaf lobes, and color of root pulp.

When examining historical characterization descriptors individually, we observed that among all cases, only four MLGs (cases 5, 6, 7, and 8) had accessions with the same shape of the central leaf, either lanceolate or ovoid (**Table 6**; **Figure 6**). In all other instances, there were varied records for the central leaf shape within an MLG. Case 16, for example, encompasses 12 accessions, 10 of them documented with distinct central leaf shapes like oblong-lanceolate, linear-pandurate, lanceolate, and straight or linear (**Supplementary Table 5**, and **Table 6**). Note that cases where the MLG had only one accession with information for a particular descriptor were not counted as ones. In terms of petiole color, accessions within three MLGs (cases 1, 8, and 9) shared the same petiole color, while all other MLGs featured accessions with multiple petiole colors (green, green with some red, purple, red, red with some green or yellowish-green). **Figure 6** showcases two accessions from case 16: AGR79 with linear-pandurate lobes and a red petiole, and BRA1193 with lanceolate central lobes and a yellowish-green petiole record. Regarding the color of the first expanded leaf and the number of leaf lobes, there were 10 and 9 cases, respectively, where the color and the number of lobules were consistent. For instance, cases 8 and 9 exhibited an equal number of leaf lobules. However, cases 1, 14, 16, and 19 contained accessions with varying numbers of lobes (**Figure 6**). Finally, among the cases examined, 13 showed consistency in the color of the root pulp, including instances like case 9 with accessions GUA80 and GUA89 (**Figure 6**). However, seven cases exhibited variations in the records for root pulp color. For example, case 20 consisted of 84 accessions, including BRA1031 and BRA265, which displayed differences in pulp color (**Figure 6**).

The examination of cases across the four passport variables and five historical characterization descriptors revealed that none of the 20 cases showed concordance across all nine reviewed descriptors. Nine cases had incomplete records, marked as “ND” (not determined), with the absence of information varying from 1 to 4 instances. Among these 20 cases, the highest number of coincidences observed was 7, found in only 3 MLGs (cases 2, 7, and 9). The remaining cases of MLGs with genetically redundant accessions showed coincidences of− 16 variables. Interestingly, the multi-accession MLGs with 84 accessions had zero coincidences across the nine variables under review (**Table 6**). The complete-linkage hierarchical agglomerative clustering of cases 1, 8, 9, 14, 19, and 20 is shown in **Supplementary Figure 5A**, displaying similarities in the clustering between SNPs and SilicoDArTs. Similarly, as observed in **Figures 4B**, **3** or **5** out of the 20 cases of SNPs and SilicoDArTs, respectively, displayed genetic distances across some of accession pairs higher than the given threshold (**Supplementary Figure 5B**).

## 4 Discussion

Cassava is currently the third-largest source of carbohydrates for human consumption in the world after rice and maize, playing a crucial role in ensuring food security, particularly in many low-income countries (Howeler et al., 2013). Global cassava production exhibits significant yields in Africa (64.8%), Asia (26.9%), and Latin America (8.3%) (FAOSTAT, 2021), with a total production of 314 million tons in 2021.While productivity is influenced by factors such as agronomic practices, climate, and pest management, major challenges include susceptibility to pests and diseases, post-harvest losses, and the pressing need for improved varieties with higher yields and nutrition content (Montagnac et al., 2009; Zainuddin et al., 2018; Ntui et al., 2023).

To address these challenges, genebanks play a crucial role in conserving cassava diversity, serving as a foundation for breeding programs and facilitating the identification of sources of resistance to pests and diseases (Bellotti and Arias, 2001; Sheat et al., 2019). The operational procedures for seed storage and plant propagation have been in place for decades, enabling genebanks to establish, maintain, and conserve collections of plant genetic resources for crop improvement (Mascher et al., 2019). Nowadays, genebanks prioritize acquiring knowledge about their existing collections, focusing on enhancing the efficiency of genetic resources management and enhancing utilization, rather than expanding their collections (van Treuren and van Hintum, 2003). Research focused on comprehending and optimizing the composition of collections is therefore of particular interest (Nadeem et al., 2018; Mascher et al., 2019; Milner et al., 2019; Sansaloni et al., 2020). To optimize collection composition, it is essential for genebanks to prioritize the identification and elimination of redundancies, thereby considering both genetic and economic perspectives (van Treuren and van Hintum, 2003). Determining the appropriate thresholds to declare two genotypes as identical presents a challenging decision. Previous studies investigating genetic redundancy in cassava collections have defined their minimum genetic distance at 0.05, either arbitrarily (Albuquerque et al., 2019) or empirically (Orek et al., 2023; Soro et al., 2023). Empirical definitions have used the distribution of pairwise distances between duplicated DNAs as a ‘calibration principle’ (Noli et al., 2013; Rabbi et al., 2015). In our study, we took an empirical approach, optimizing the thresholds for identifying genetically redundant accessions at 0.015 and 0.025 genetic distance for SNP and SilicoDArT markers, respectively (**Table 3**; **Figure 3**). To achieve this, we utilized a subset comprising 21 accessions from the core collection, along with technical and biological replicates. DNA-Reps were employed to reduce potential miscalling errors during genotyping, specifically for some heterozygous SNPs misidentified as homozygotes due to low sequencing read depth (Hamblin and Rabbi, 2014). Extract-Reps were used to address any traceability or contamination errors during DNA extraction. Additionally, Ind-Reps were considered to account for biological differences, potentially arising from traceability errors during routine multiplication of *in-vitro* plantlets.

The use of exemplary quality parameters for marker selection and sample exclusion had a notable impact on the estimated genetic distances (IBS and Jaccard) among replicates, resulting in reduced mean and standard deviation values (**Table 2**; **Figures 1A**, **2**). Parameters such as call rate and maf were instrumental in selecting high-informative markers with minimal missing data (above 20 percent) and minor allele frequency greater than 0.001. Additionally, the choice of markers based on the average number of sequence tag copies (AvgMarkerCount) facilitated the removal of markers with insufficient sequencing depth. Furthermore, markers were selected with a low coefficient of variation of AvgMarkerCount (CVMarkerCount) to exclude potential paralog sequences (McKinney et al., 2017). Crucially, sample quality played an important role in optimizing the minimum genetic distances for identifying redundant genotypes (**Supplementary Figure 2**; **Table 3**). Target QC is crucial for sequencing library suitability. While non-”good” categories may sometimes result in lower read counts, it is not always the case (**Figure 2**). Therefore, considering multiple sample quality parameters is vital for selecting high-quality samples. Ignoring this can overestimate genetic distances for redundant accessions (**Supplementary Figure 2**). This is evident in individual SNP call rate, showing a tendency for lower values, and in reviewing individual Ho and OneRatio of SilicoDArT markers (**Figure 2**). Low individual Ho for SNP and OneRatio of SilicoDArT marker values may result from low call rates, possibly due to low total read counts, while high values for these two parameters may indicate unintended DNA sample cross-contamination. However, caution is needed, as these parameters, if not used carefully, may introduce bias and eliminate samples with exceptional individual Ho and OneRatio values due to genetic background. Excluding samples with exceptionally low counts in total sequenced tags (tagcounttotal), unique reads (tagtotalunique), individual SNP call rate, individual Ho, and individual OneRatio (the latter for SilicoDArTs) proved effective in eliminating samples with probable technical issues, primarily derived from low-quality DNA or cross-contamination. Most of these problematic samples were identified as downshifted libraries. By implementing this set of parameters, it was possible to significantly reduce the minimum genetic distance thresholds, leading to the consolidation of all replicates from a single accession within individual MLGs.

The identification of MLGs with SNP markers has been employed in studies to assess genetic redundancy within cassava collections (Albuquerque et al., 2019; Soro et al., 2023). MLGs represent the combination of alleles at multiple genetic loci within an individual or group of individuals. This approach can be used to identify genetic redundancy (potential duplicate accessions) and to determine genetic distinctness (unique accessions) within a collection. In our study, we employed MLG detection to identify genetic distinctness and redundancy within CIAT’s cassava collection, comprising 5,302 accessions. We utilized two marker types: co-dominant SNPs and dominant SilicoDArT markers and compared results across the two approaches. The dominant markers, provide binary information, indicating the presence or absence of a specific allele at a particular locus, while the codominant markers provide more detailed information by distinguishing between different genotypes/allele combinations at a specific locus (Amiteye, 2021). The resulting genetic distance matrices of each genetic marker, IBS and Jaccard, exhibited high similarity in the number of MLGs detected, with 2,518 (47.4 percent) and 2,526 (47.6 percent) MLGs out of the 5,302 accessions, respectively (**Table 4**). Redundancy was observed across the five regions from which germplasm originates, but it was notably higher in accessions from Central/North America & the Caribbean (**Table 5**; **Supplementary Figure 4**). A total of 374 accessions exhibited discrepancies between both methods, with around 127 accessions being identified as unique by one method and redundant by the other. These accessions need to be further reviewed, especially in the cases where accessions have been detected as unique by at least one approach. Interestingly, upon examining the dispersions of genetic distances for various MLG sizes, it becomes evident that for certain pairs of accessions within multi-accession MLGs of different sizes, the distance exceeds the minimum genetic distance thresholds used (**Figure 4B**).

In this study, we do not delve into the population structure of the collection. Instead, the focus of the hierarchical clustering analysis conducted on the 5,302 accessions is to compare and visualize the levels of genetic redundancy within and across regions of origin, as well as across marker types. A more comprehensive analysis, conducted in collaboration with the cassava collection conserved by the International Institute for Tropical Agriculture (IITA), is currently underway as a separate study. A recent study by Perez-Fon et al. (2023) identified two main gene pools, North & Northwest of the Amazon River basin (ARB) and South & Southeast of ARB, when assessing the genetic diversity of a set of 481 accessions selected as the most heterogeneous and unique cassava landraces. Without conducting additional analysis, our hierarchical clustering analysis using IBS (SNP) and Jaccard (SilicoDArT) distances revealed the presence of at least three major groups (**Supplementary Figure 4**). The major group predominantly comprises accessions from western South America and Central/North America & Caribbean. A second group encompasses a blend of accessions from eastern South America, western South America, Asia, and Africa. Finally, a third group, predominantly comprising accessions from eastern South America, was identified (**Table 1**; **Supplementary Figure 4**).

While genomic information proves valuable for assessing genetic redundancy, interpreting molecular data remains complex due to the presence of diverse genetic relationships among potential duplicates (van Treuren and van Hintum, 2003). To complement the MLG analysis results, especially for identified redundant groups across both SNP and SilicoDArT approaches, we reviewed and compared additional information from passport data and available historical characterization records. Passport data includes essential details such as genus name, country of origin, acquisition date, unique accession number, among others. Characterization data provides in-depth descriptions of plant germplasm, serving as a tool to confirm their authenticity and identify duplicates in a collection. Cassava experts have agreed upon a set of descriptors for characterization (Bioversity Int and CIAT, 2009) including shape of central leaf, petiole color, color of the first expanded leaf, number of leaf lobes, and color of root pulp.

We selected 20 multi-accession MLGs with varying number of accessions (ranging from 2 to 84) to review the passport and historical characterization data. Discrepancies and agreements were observed among accessions collapsed within multi-accession MLGs (**Table 6**; **Figure 6**). These differences can be attributed to various reasons. The five characterization descriptors compared were documented between 10 and 20 years ago, sourced either from the in-field collection maintained until the 1990s or from breeding programs as part of specific projects in the last decade (Mafla et al., 1993). Unfortunately, there is a gap in the information about the source of the five reviewed descriptors from characterization data, although it is known that records were collected before 2002 and root and leaf images were uploaded to the cassava database during 2002 and 2009/2013, respectively. These records may not directly align with the genotyped accessions, implying possible tracking errors during multiple multiplication cycles.

Furthermore, some multi-accession MLGs included accessions with genetic distances higher than the designated thresholds. This is evident from certain accession pairs that exceed the specified genetic threshold for MLG identification (**Figure 4B**; **Supplementary Figure 5**), indicating that MLGs with greater dispersion of genetic distances require further inspection. On the other hand, a major obstacle to validating the genetic redundancy of MLGs with the characterization data is its scarcity and incompleteness. Among the five characterization descriptors, missingness ranged from 23 to 80 percent. Hence, our preference is to categorize these groups of accessions, or multi-accession MLGs, as redundant rather than as duplicates, until resources become available to facilitate side-by-side comparison in the field. We consider three possible sources of error that can lead to the MLG clusters not corresponding to the passport and characterization data: (i) potential errors in the traceability of phenotypic data, (ii) potential cumulative errors as a result of decades of *in-vitro* propagation, before implementing the use of barcodes, and (iii) potential errors in the traceability of samples during the DNA extraction and genotyping process.

Considering the high cost of maintaining and distributing cassava collections — estimated at US$71 USD per accession/year in 2011 (CGIAR Genebanks Consortium, 2011) that would now the equivalent of US$97 per accession — identifying duplicates within the collection is crucial for optimizing physical storage space, reducing maintenance costs, enhancing characterization, and ensuring collections’ accessibility and usability. Duplicates within a collection may arise by mistake when a variety is introduced in the collection more than once for various reasons, such as different names given to the same varieties by farmers, and/or by inadequate documentation or record-keeping practices resulting in the same variety being catalogued multiple times, among other factors. Furthermore, the process of multiplying accessions over a span of more than 40 years may have caused mixing, leading to duplications under different accession names. This implies that the original diversity preserved in the genebank may have been compromised, to some extent, from the lack of methods ensuring traceability some decades ago.

The identification of distinct accessions is also relevant for managing the collections, for planning for new initiatives within genebanks, and for facilitating access and characterization. Cryopreservation of vegetatively propagated germplasm becomes a viable option for base collections as new techniques are developed (Jenderek and Reed, 2017). Current efforts are underway to establish a cryo-collection at CIAT’s genebank. The 1,440 distinct accessions identified through SNP and SilicoDArT markers are primary candidates to initiate this process. Similarly, some of the multi-accession MLGs that collapsed accessions falling below the used genetic thresholds are most likely duplicates. On the other hand, there is a need to further revise cases of multi-accession MLGs that group accessions with pairs of genetic distances higher than the set threshold. By conducting comprehensive genetic analyses, curators can make informed decisions regarding the conservation, utilization, and breeding of plant varieties. This information helps in identifying unique traits, understanding evolutionary relationships, and ensuring the preservation of valuable genetic material for future agricultural needs. Consequently, this study offers valuable insights for genebank curators, enabling them to (i) identify a genetically distinct subset of accessions for targeted cryopreservation efforts, and (ii) discern genetic redundancy and potential accession duplicates. This information not only facilitates follow-up studies but also opens the door for potential removal of duplication from the collection, thereby reducing conservation costs and enhancing accessibility to cassava diversity.

## Data availability statement

The datasets presented in this study can be found in online repositories. The names of the repository/repositories and accession number(s) can be found below: https://dataverse.harvard.edu/privateurl.xhtml?token=cd06716b-4004-4918-9cb3-4030c39f97ce, Aliance Bioversity Int. and CIAT Dataverse repository and https://gigwa.cgiar.org/FutureSeeds/?module=Cassava_study, Genotype Investigator for Genome-Wide Analyses (Gigwa).

## Author contributions

MC-Y: Conceptualization, Data curation, Formal analysis, Methodology, Writing – original draft. JO: Methodology, Writing – review & editing. EA: Methodology, Writing – review & editing. MV-T: Methodology, Writing – review & editing. MC: Visualization, Writing – review & editing. NM: Writing – review & editing. PW: Conceptualization, Funding acquisition, Methodology, Writing – review & editing.
