# Supplementary material for: Identifying genetically redundant accessions in the world’s largest cassava collection
Source: Front Plant Sci. 2024 Jan 18;14:1338377. doi: 10.3389/fpls.2023.1338377 (PMC10830726; doi:10.3389/fpls.2023.1338377)
Supplement: Supplementary file 1 [file DataSheet_1.pdf]

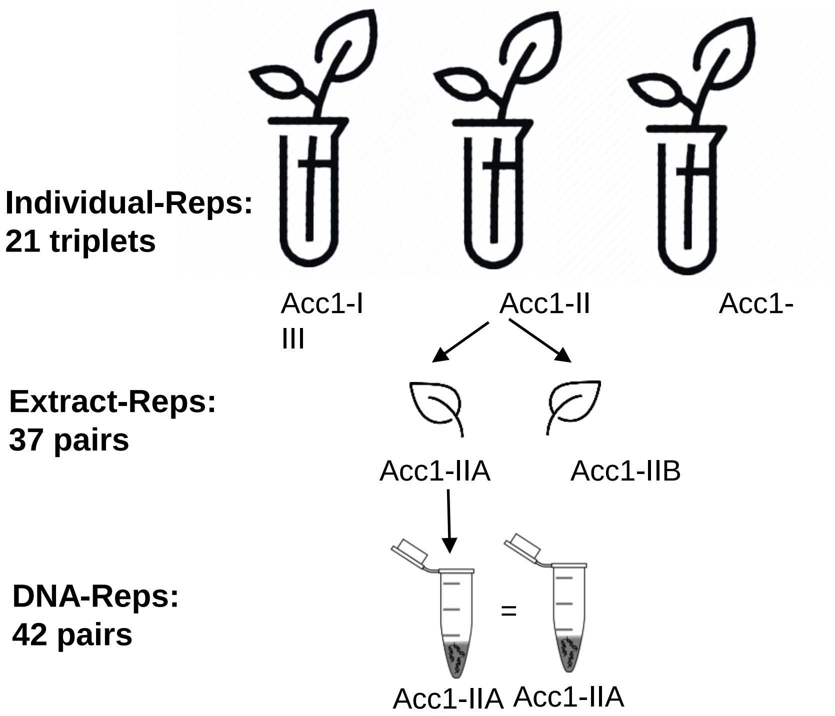

Supplementary Figure 1. Twenty-one cassava (*M. esculenta*) accessions from the CIAT core collection were selected to produce three types of replicates (i) different individuals from the same accession "Individual-Reps", (ii) different DNA samples extracted from the same individual "Extract-Reps", and (iii) the same DNA sample analyzed twice "DNA-Reps".

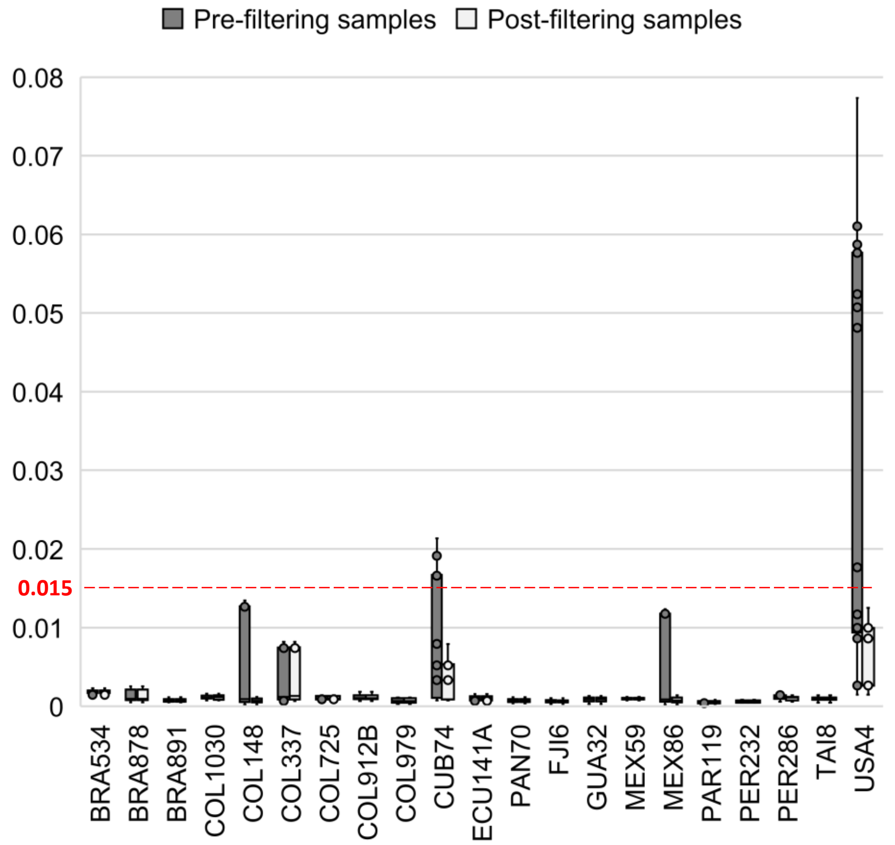

Supplementary Figure 2. Boxplot illustrating the distribution of genetic distances per accession for pre-filtered and post-filtered samples. The dashed red line shows the new allowed threshold of 0.015 with the SNPs dataset.

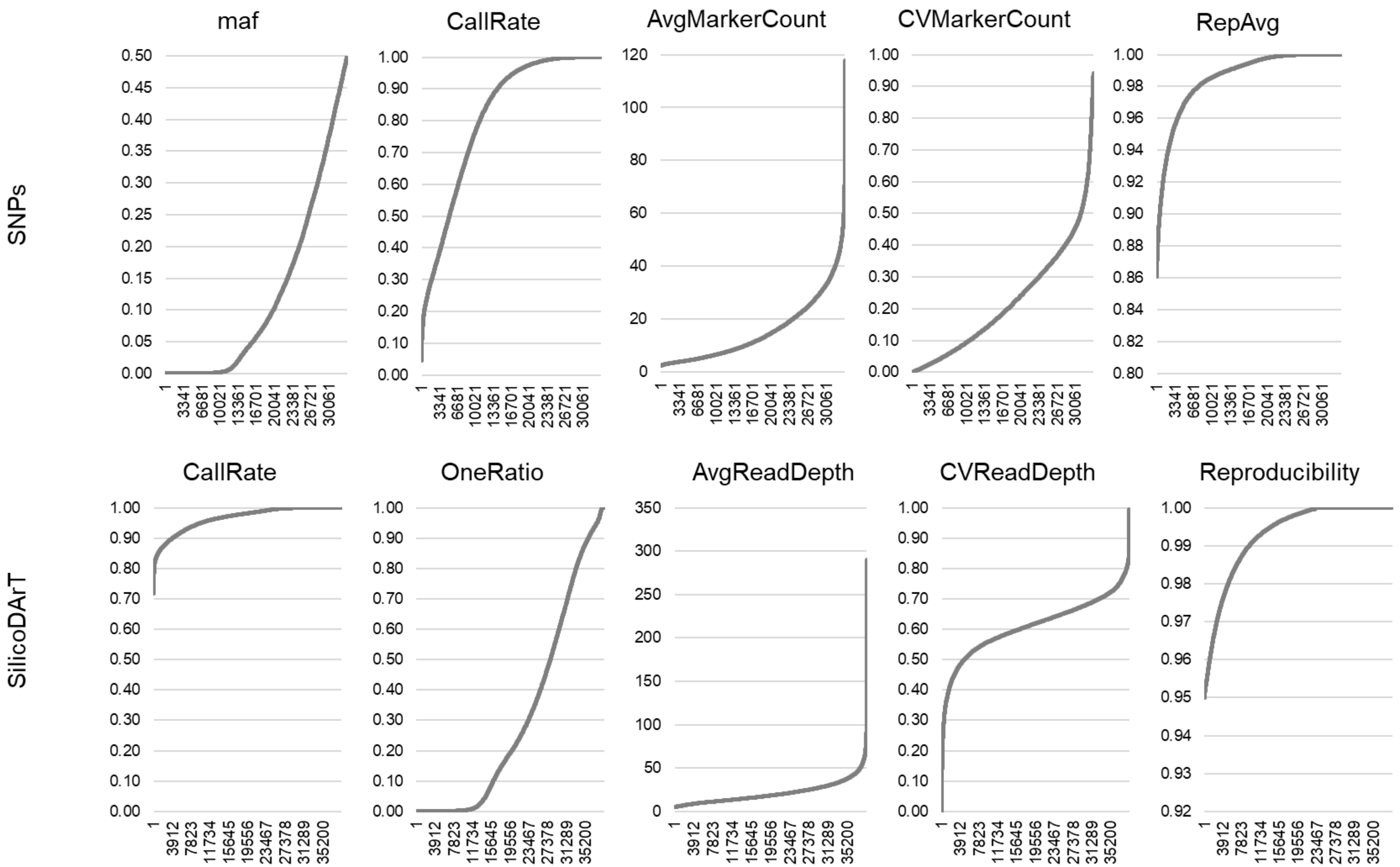

Supplementary Figure 3. Plots illustrating the distribution of the used marker-quality parameters across the 5,302 accessions and 33,395 SNPs (upper) or 39,103 SilicoDArT markers (lower).

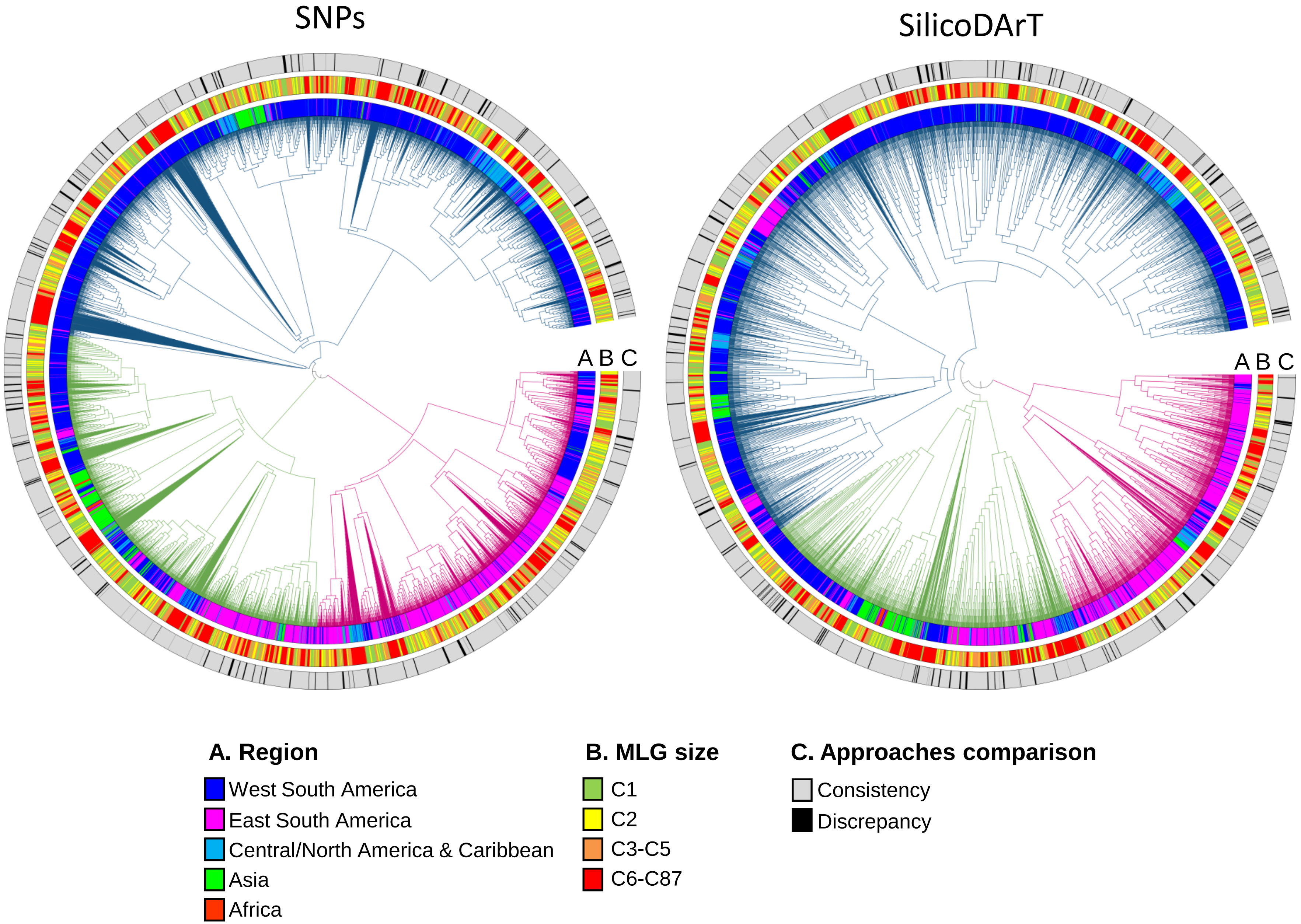

Supplementary Figure 4. Clustering patterns of 5,302 accessions compared to genetic redundancy patterns across diverse regions and marker types. Dendrograms resulting from ward.D2 linkage method analysis using IBS (SNPs; left) and Jaccard (SilicoDArT; right) distances is presented. Cluster 1 (pink) mainly includes East South American accessions, Cluster 2 (green) consists of diverse accessions from East and West South America, Asia, and Africa, and Cluster 3 (blue) primarily features West South American accessions, with some from Central/North America and the Caribbean. Around dendrograms, accessions are color-coded by region (A), MLG size categories (B), and discrepancies in MLGs detection between SNPs and SilicoDArT (C).

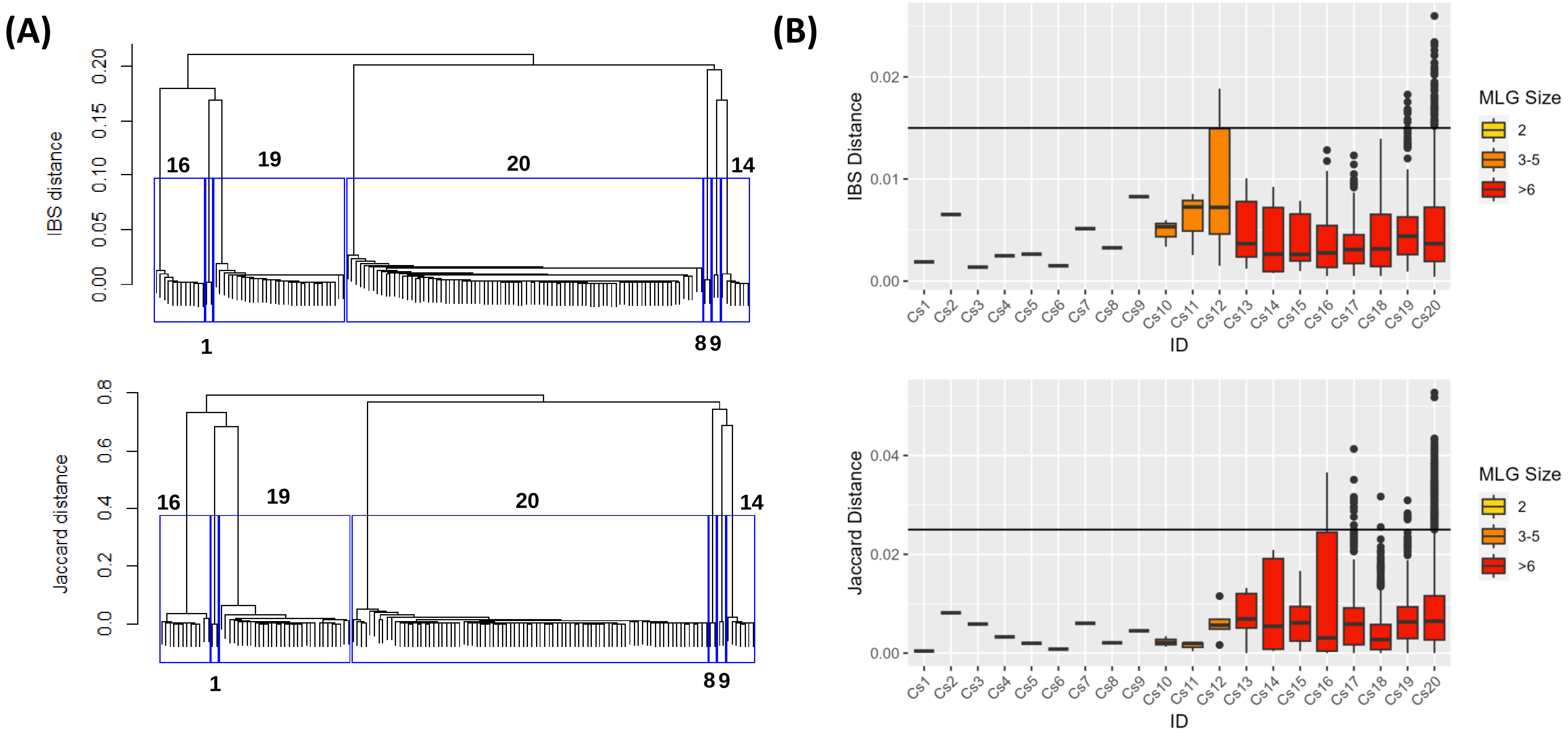

Supplementary Figure 5. (**A**) Complete-linkage hierarchical agglomerative clustering of cases 1, 8,9,14,16,19,20 from multi-accession MLGs prepared using IBS (upper) and Jaccard (lower) genetic distances. (**B**) Dispersion of genetic distances among pairs of accessions within multi-accession MLGs of varying group sizes, cases 1 to 20. The upper boxplot displays IBS distances estimated from SNPs. The lower boxplot shows Jaccard distances estimated from SilicoDArT markers. Group sizes are categorized and represented in different colors. Thresholds 0.015 and 0.025 are shown with a black line.

Supplementary Table 1. Summary of accessions and the number of technical and biological replicates utilized in this study. Several samples served multiple purposes, leading to varying numbers of replicates per accession. The total samples per accession are indicated below

Accession	DNA-Reps		Extract-Reps		Individual-Reps		Country	All samples
	samples	pairs	samples	pairs	samples	trio		
BRA534	4	2	4	2	3	1	Brazil	7
BRA878	4	2	4	2	3	1	Brazil	7
BRA891	4	2	4	2	3	1	Brazil	7
COL1030	4	2	2	1	3	1	Colombia	6
COL148	4	2	4	2	3	1	Colombia	7
COL337	4	2	4	2	3	1	Colombia	7
COL725	4	2			3	1	Colombia	5
COL912B	4	2	4	2	3	1	Colombia	7
COL979	4	2	4	2	3	1	Colombia	7
CUB74	4	2	4	2	3	1	Cuba	7
ECU141A	4	2	4	2	3	1	Ecuador	7
FJI6	4	2	4	2	3	1	Fiji	7
GUA32	4	2	4	2	3	1	Guatemala	7
MEX59	4	2	4	2	3	1	Mexico	7
MEX86	4	2	4	2	3	1	Mexico	7
PAN70	4	2	4	2	3	1	Panama	7
PAR119	4	2	2	1	3	1	Paraguay	6
PER232	4	2	2	1	2	1	Peru	5
PER286	4	2	4	2	3	1	Peru	7
TAI8	4	2	4	2	3	1	Thailand	7
USA4	4	2	4	2	3	1	USA	7
**Total**	**84**	**42**	**74**	**37**	**62**	**21**		**141**

Supplementary Table 2. Statistical summary of sample-quality parameters across 5,555 samples and the thresholds used to select high-quality samples. Number of total samples below parameter threshold are shown.

Quality parameter	Min	Q1	median	mean	Q3	Max	Thershold	Samples below threshold
Tagcounttotal	1007722	2160390	2296789	2306247	2475498	3995430	> 1,500,00	7
Tagcountunique	92395	349765	382200	385875	414168	1136722	> 230,000	27
Individual SNP callrate	0.110	0.800	0.810	0.811	0.830	0.910	> 0.73	72
Individual observed heterozygosity (Ho)	0.000	0.080	0.090	0.088	0.090	0.200	> 0.05 & < 0.16	79
Individual SilicoDArT callrate	0.865	0.962	0.970	0.969	0.978	1.000	> 0.996	30
Individual SilicoDArT OneRation	0.040	0.270	0.270	0.273	0.280	0.350	< 0.2	21

Supplementary Table 3. Summary of samples that either failed or passed all the 6 quality parameters (QP) reviewed and listed in Supplementary Table 2.

Number of failed QP.	Target QC			Total
	good	downshifted	weak	
0	5407	3	2	**5412**
1	99	2	0	101
2	14	2	0	16
3	9	9	0	18
4	3	0	0	3
5	0	3	0	3
6	0	2	0	2
Number of replicated samples that failed to QP	10	0	0	10
Number of non-replicated samples that failed QP	115	18	0	133
**Number of removed samples from the analysis**	**125**	**18**	**0**	**143**
Number of total samples assessed in this study	5532	21	2	5555
**Number of unique accessions retained for MLG analysis**				**5302**

Supplementary Table 4. Statistical summary of marker-quality parameters across 5,302 and the thresholds used to select high-quality SNP and SilicoDArT markers. Number of total markers retained for subsequent analysis are shown.

Marker	Parameter	Min	Q1	median	mean	Q3	Max	Threshold	SNPs number retained	(%) SPNs retained
SNPs	no filters applied	–	–	–	–	–	–	–	33,395	100
	maf	0.00009	0.00094	0.05789	0.12282	0.21191	0.50000	≥ 0.001	24,915	74.6
	Callrate	0.04413	0.68446	0.94549	0.81456	0.99566	1.00000	≥ 0.8	16,962	50.8
	AvgMarkerCount	2.51000	5.84600	11.32000	15.49900	21.93000	117.66800	≥ 12	8,959	26.8
	CVMarkerCount	0.00001	0.07491	0.18578	0.22167	0.33266	0.94230	≤ 0.6	8,582	25.7
	RepAvg	0.86080	0.98240	0.99460	0.98540	0.99980	1.00000	≥ 0.98	7,890	23.6
	Mapped to genome Refv7	–	–	–	–	–	–	–	7,180	21.5
SilicoDArT	no filters applied	–	–	–	–	–	–	–	39,103	100
	Callrate	0.71690	0.95060	0.98230	0.96650	0.99940	1.00000	≥ 0.95	29,411	75.2
	OneRatio	0.00019	0.00321	0.19315	0.31374	0.58017	0.99981	≥ 0.05	14,990	38.3
	AvgReadDepth	5.00100	12.33300	18.36400	21.53200	27.35600	289.92800	≥ 12	10,171	26.0
	CVReadDepth	0.00000	0.55930	0.61950	0.60940	0.67530	0.99500	≤ 0.7	8,321	21.3
	RepAvg	0.95000	0.99060	0.99840	0.99290	1.00000	1.00000	≥ 0.98	8,186	20.9

Supplementary Table 5. Descriptors of passport and characterization data of selected 20 cases of MLGs with varying group size. The number of accessions with the specific descriptors is provided. Not determined (ND)

Descriptor	Descriptor level	Case	1	2	3	4	5	6	7	8	9	10	11	12	13	14	15	16	17	18	19	20
		**MLG**	**4**	**5**	**13**	**25**	**37**	**42**	**47**	**49**	**207**	**11**	**35**	**16**	**2468**	**393**	**2525**	**1168**	**1182**	**2313**	**739**	**4535**
		**size**	2	2	2	2	2	2	2	2	2	3	3	4	6	7	7	12	28	29	31	84
ShapeCentralLeaf	Elliptic-Lanceolate		1				1					2	1	2	1		1		3	1		6
	Lanceolate		1	2	1	1	1	2	2		1	1	2	1	5	5	5	3	22	8	3	69
	Linear-pandurate																	2	1		3	1
	Linear-Piramidal																1					1
	Oblong-Lanceolate										1							1				
	Obovate-Lanceolate				1															15		
	Ovoid									2										1		
	Pandurate																			2	1	
	Straight or linear													1		1		4			11	
	**Grand Total**		**2**	**2**	**2**	**1**	**2**	**2**	**2**	**2**	**2**	**3**	**3**	**4**	**6**	**6**	**7**	**10**	**26**	**27**	**18**	**77**
	**Number levels/MLG**		**2**	**1**	**2**	**1**	**2**	**1**	**1**	**1**	**2**	**2**	**2**	**3**	**2**	**2**	**3**	**4**	**3**	**5**	**4**	**4**
PetioleColor	Green								1		2		2			2			2	1		
	Green with little red																					1
	Purple																	1			1	
	Red														2		1	5		4	5	1
	Red with little green		2	1	1					2			1	2	1	1		1		2	1	
	Yellowish Green													2				1	1			2
	**Grand Total**		**2**	**1**	**1**				**1**	**2**	**2**		**3**	**4**	**3**	**3**	**1**	**8**	**3**	**7**	**7**	**4**
	Number levels/MLG		1	1	1	ND	ND	ND	1	1	1	ND	2	2	2	2	1	4	2	3	3	3
Color1stExpandedLeaf	dark green		2	1	1					2	2		3	3	3	3	1	6	3	7	7	3
	light green								1					1				1				1
	**Grand Total**		**2**	**1**	**1**				**1**	**2**	**2**		**3**	**4**	**3**	**3**	**1**	**7**	**3**	**7**	**7**	**4**
	Number levels/MLG		1	1	1	ND	ND	ND	1	1	1	ND	1	2	1	1	1	2	1	1	1	2
NumberLeafLobes	five		1							1					2			2	1	3	4	
	nine								1													2
	seven		1	1	1					1	2		3	4	1	2	1	5	2	2	1	2
	three															1						
	**Grand Total**		**2**	**1**	**1**				**1**	**2**	**2**		**3**	**4**	**3**	**3**	**1**	**7**	**3**	**5**	**5**	**4**
	Number levels/MLG		2	1	1	ND	ND	ND	1	2	1	ND	1	1	1	2	1	1	1	1	1	2
ColorRootPulp	Cream						2							1		4						54
	White		2	2	2	1		2	2		1	3	2	3	5	1	7	7	25	26	11	18
	Yellow												1			1						5
	**Grand Total**		**2**	**2**	**2**	**1**	**2**	**2**	**2**		**1**	**3**	**3**	**4**	**5**	**6**	**7**	**7**	**25**	**26**	**11**	**77**
	Number levels/MLG		1	1	1	1	1	1	1	ND	1	1	2	2	1	3	1	1	1	1	1	3
BiologicalStatus	BreedersLine				1	1	1						3	3	1	1				2	3	1
	Landrace		2	2	1	1	1	2	2	2	2	3		1	5	6	7	12	28	27	28	83
	**Grand Total**		**2**	**2**	**2**	**2**	**2**	**2**	**2**	**2**	**2**	**3**	**3**	**4**	**6**	**7**	**7**	**12**	**28**	**29**	**31**	**84**
	Number levels/MLG		1	1	2	2	2	1	1	1	1	1	1	2	2	2	1	1	2	2	2	2
CountriesOrigin	ARG																	2		1	16	
	BRA		2		1		1	2	2	2					2	1	5	9		14	8	3
	**CHN**																1					
	COL				1								3	4		6			28	10	4	78
	CRI																				1	
	**CUB**																			1		
	ECU																					1
	GTM										2											
	**MEX**														1							
	MYS			2																		
	PAN														1							
	**PER**					1						3					1	1				1
	PRY														1					1	2	
	THA					1																
	**VEN**						1								1					2		1
	**Grand Total**		**2**	**2**	**2**	**2**	**2**	**2**	**2**	**2**	**2**	**3**	**3**	**4**	**6**	**7**	**7**	**12**	**28**	**29**	**31**	**84**
	Number levels/MLG		1	1	2	2	2	1	1	1	1	1	1	1	5	2	3	3	1	6	5	5
